# A Multi-Objective Demand Response Optimization Model for Scheduling Loads in a Home Energy Management System

**DOI:** 10.3390/s18103207

**Published:** 2018-09-22

**Authors:** Jaclason M. Veras, Igor Rafael S. Silva, Plácido R. Pinheiro, Ricardo A. L. Rabêlo, Artur Felipe S. Veloso, Fábbio Anderson S. Borges, Joel J. P. C. Rodrigues

**Affiliations:** 1 Graduate Program in Applied Informatics, University of Fortaleza (UNIFOR), Fortaleza-CE 60811-905, Brazil; jaclason@ufpi.edu.br (J.M.V.); placidrp@gmail.com (P.R.P.); 2 Department of Computing, Federal University of Piauí (UFPI), Teresina-PI 64049-550, Brazil; yggor14rafa@hotmail.com (I.R.S.S.); arturfdasveloso@gmail.com (A.F.S.V.); fabbioanderson@gmail.com (F.A.S.B.); 3 National Institute of Telecommunications (INATEL), Av. João de Camargo, 510-Centro, Santa Rita do Sapucaí-MG 37540-000, Brazil; joeljr@ieee.org; 4 Instituto de Telecomunicações, 1049-001 Lisboa, Portugal; 5 Institute Photonics and Optoinformatics, University of Information Technology, Mechanics and Optics (ITMO), 197101 Saint Petersburg, Russia

**Keywords:** demand response, energy management, load scheduling, multi-objective optimization

## Abstract

Demand Response (DR) aims to motivate end consumers to change their energy consumption patterns in response to changes in electricity prices or when the reliability of the electrical power system (EPS) is compromised. Most of the proposals found in the literature only aim at reducing the cost for end consumers. However, this article proposes a home energy management system (HEMS) that aims to schedule the use of each home appliance based on the price of electricity in real-time (RTP) and on the consumer satisfaction/comfort level in order to guarantee the stability and the safety of the EPS. Thus, this paper presents a multi-objective DR optimization model which was formulated as a multi-objective nonlinear programming problem subjected to a set of constraints and was solved using the Non-Dominated Sorted Genetic Algorithm (NSGA-II), in order to determine the scheduling of home appliances for the time horizon. The multi-objective DR optimization model not only to minimize the cost of electricity consumption but also to reduce the level of inconvenience for residential consumers. Moreover, a priori, it is expected to obtain a more uniform demand with fewer peaks in the system and, potentially, achieving a more reliable and safer EPS operation. Thus, the energy management controller (EMC) within the HEMS determines an optimized schedule for each home appliance through the multi-objective DR model presented in this article, and ensures a more economic scenario for end consumers. In this paper, a performance evaluation of HEMS in 15 Brazilian families between 1 January and 31 December 2016 is presented with different electric energy consumption patterns in the cities of Belém—PA, Teresina—PI, Cuiabá—MT, Florianópolis—SC and São Paulo—SP, with three families per city, located in the regions north, northeast, central west, south and the southeast of Brazil, respectively. In addition, a total of 425 home appliances were used in the simulations. The results show that the HEMS achieved reductions in the cost of electricity for all the Scenarios used while minimally affecting the satisfaction/comfort of the end consumers as well as taking into account all the restrictions. The largest reduction in the total cost of electricity occurred for the couple without children, resident in the city of Teresina—PI; with a drop from US$ 99.31 to US$ 90.72 totaling 8.65% savings in the electricity bill. Therefore, the results confirm that the proposed HEMS effectively improves the operating efficiency of home appliances and reduces electricity costs for end consumers.

## 1. Introduction

The increase of the global population has caused a greater complexity of the electricity supply. Due to this, there is a need for studies and research concerning the quality and reliability of electric power systems in order to avoid interruptions in the supply of electricity and in price increases, among other problems [1,2,3,4]. At the same time, the pressure on natural resources worldwide and concern for the environment is also increasing rapidly. One of the solutions to help overcome such problems is to use a smart grid (SG). An SG is a system that applies information and communication technologies (ICT) to improve the interaction between all the devices of an electrical power system (EPS) and consumers connected to it [5]. This interaction can be used by end consumers to improve their electricity consumption pattern in order to reduce the cost of electricity.

The authors in [6] state that the demand response control methodologies and smart appliances can optimize the use of electrical resources more efficiently. In this sense, the authors in [7,8,9] defined a demand response (DR), from the point of view of a smart grid, as a program that provides various incentives and benefits to end consumers to change their electricity consumption patterns in response to changes in the price of electricity over time or when electrical power network reliability is compromised by any EPS overhead.

The most commonly used DR programs (DRPs) are based on price, following one of three tariff models: (1) Time-of-Use (TOU), which offers consumers different electric energy tariffs during different periods of the day [10,11] and is generally based on the average cost of generation and delivery of energy over a 24-h period [12]; (2) Real-Time Pricing (RTP), when the price of electricity is modified hourly throughout the day, and this may reflect the cost for generation or the wholesale price level; and finally, (3) Critical-Peak Pricing (CPP), which is a dynamic pricing mechanism that uses elements of TOU and RTP to adjust tariffs as a temporary response to events or conditions such as high market prices, or decreasing reserves [10]. The authors in [5,13] affirm that RTP has a much greater flexibility than TOU and CPP. Therefore, the increase in the price of the tariff is linked to the increase in demand for electricity or the low energy productivity of the EPS.

Thus, the DRPs can be regarded as one of the most important tools for Home Energy Management Systems (HEMS). DRPs are able to interrupt, control, regulate, or curtail the energy of the devices and end consumers have financial support to modify their electricity consumption patterns in order to improve the reliability and efficiency of EPS [14]. Moreover, DRPs help the utility companies to shift the load from peak hours to off-peak hours in order to reduce electricity prices as well as to balance the supply and demand [15].

Due to the costs and restrictions related to energy, HEMS is of great importance nowadays because it is becoming essential for modern societies, cities, and smart homes [16,17]. HEMS manages home energy consumption in order to increase the stability and efficiency of the EPS using Internet of Things (IoT) and optimization algorithms. The authors in [14] describe IoT as a technological revolution in terms of information and communication. IoT allows Radio Frequency Identification (RFID) tags, sensors, actuators, smartphones, etc. into a network where they are able to inter-communicate without human intervention for a common purpose. The IoT has introduced fresh applications, i.e., smart homes, smart cities and so on. Therefore, different techniques are being studied to improve residential energy usage. The main technique to improve energy usage is by adjusting the planning of residential appliances to maximize the consumption. Such adjustments allow a reduction in the final amount of energy required and, by operating the appliances in periods when the cost of electricity is lower, reduce the final costs even further; moreover, the use of appliances in off-peak hours with cheaper rates reduces the demand during peak-hours [14,15].

End consumers have home appliances [18,19,20] that need to be programmed in an orderly manner to guarantee a balance between supply and demand of electric energy [18,20,21]. However, the programming of these home appliances within the same time interval requires specific knowledge and availability of time on the part of the consumer [22]. In addition, residential management scheduling must take into account consumer preferences regarding the usage of these appliances and the price variation of electricity. Consequently, an infrastructure able to program the operational periods of these home appliances over the planning horizon is required. This program must be able to adjust itself in relation to the peak periods, and thus improve the reliability and efficiency of the EPS without modifying the satisfaction/comfort of the consumers. Although problems of DR in smart grid environments have been investigated in recent studies [23,24,25,26,27], the scheduling of residential loads considering the different peculiarities that involve the communication system, the operating characteristics of the different categories of home appliances and the level of satisfaction/comfort of the end consumers have not been well analyzed.

This paper proposes the general architecture of an HEMS and presents a mathematically formulated multi-objective DR optimization model as a nonlinear programming (NLP) problem to determine the optimal scheduling of home appliances considering real-time pricing (RTP) as well as different categories of appliance. The multi-objective DR optimization model aims to minimize the cost of energy consumption and minimally affect convenience (satisfaction/comfort) of end consumers. The main constraints are: minimum and maximum load limits for each time period; ramp limits; minimum consumption within the planning horizon; and some restrictions for the different home appliance categories. Although it is difficult to overcome the NLP problem, it was solved by applying the Non-Dominated Sorted Genetic Algorithm (NSGA-II) [28] and an optimal solution was obtained.

The main contributions of this paper are as follows:

(1) The HEMS and multi-objective DR optimization model present in this work can optimize the scheduling of different categories of home appliances considering different planning horizons and real-time pricing. Thus, with these smart tools, families can reduce the level of dissatisfaction/discomfort as well as energy costs;
(2) The DR model presented here can be set up in any country, worldwide for any energy layout;
(3) The impact of different energy consumption profiles can be analyzed considering the management of home appliances;
(4) The system takes into account various different effects on residential energy consumption, such as geographic location, different climates and temperatures, consumer preferences and the hourly price of electricity.
(5) The ability to assess any inconvenience to end consumers so they can decide whether or not to join the DR program;
(6) A statistical evaluation of the multi-objective model with NSGA-II was performed to verify its overall performance compared to a random search algorithm;
(7) The DR model can also offer greater flexibility so that end consumers can choose their preferences considering satisfaction and costs.

The rest of this paper is organized as follows. Section 2 reviews the related work on the topic; Section 3 shows the layout of the home energy management system; Section 4 presents the multi-objective DR optimization model for electricity load scheduling and the NSGA-II optimization technique; Section 5 details a case study that shows the experimental scenarios and the numerical results obtained through simulations of the HEMS using the multi-objective model to minimize the cost of electricity associated with consumption as well as the level of inconvenience of end consumers; and, finally, Section 6 explains the main contributions of this work and outlines possible future research work.

## 2. Related Work

Significant research, in recent years, has been carried out to manage home appliances in SG environments. The authors in [5] proposed an home energy management system architecture in order to minimize the cost of electricity and the peak-to-average ratio. The proposal contemplates the management of loads through DR that was formulated mathematically as a nonlinear programming problem. The optimization problem is solved using a genetic algorithm. The approach was limited to evaluation of nine home appliances; however, all of the parameters (start and stop times; time intervals between operations) of these appliances must be programmed by the consumers.

In Ref. [13], the authors proposed a constrained Particle Swarm Optimization (PSO)-based residential consumer-centric load-scheduling method. The proposal was developed a linear programming (LP) problem. The main objective of the work is to shift load profiles by home appliances as well as cut down on peak energy demands through a new constrained swarm intelligence-based residential consumer-centric demand-side management (DSM) method. The swarm intelligence, constrained PSO, is used to minimize the energy consumption cost while considering the user’s comfort and satisfaction for the implementation fo the DR. However, the proposal only evaluated the programming of nine appliances in a household. Thus, the proposal does not consider the different categories of home appliances.

The authors in [29] proposed a DR optimization model that takes into account a set of energy-related constraints to determine the optimal operation schedule for home appliances. The objective is to minimize the cost associated with energy consumption, taking into account the satisfaction and comfort of final consumers and the various constraints associated with the consumption of electric energy. The problem was formulated as nonlinear programming. The results of the computational simulations show that the optimization process by means of a Genetic Algorithm (GA) using the model proposed in this work effectively manage the different categories of appliances in the ten Brazilian households. Thus, the proposed DR model is able to reduce the cost associated with the consumption of electric energy and the level of inconvenience of the families when considering the preferences of the consumers in relation to the use of the home appliances. However, the paper does not have a multi-objective perspective and it does not use statistical techniques to analyze and validate the DR model.

In order to cut unnecessary consumption and minimize energy costs, the authors in [17] applied a management system that integrated automatic switching off with load balancing and a planning algorithm. Cost minimization was dealt with as a mixed-integer programming problem. All appliances were scheduled to a least slack time (LST) algorithm while also taking user comfort into consideration. The computational simulations showed that the LST plan reduced the costs of energy consumption. However, the different classes of appliances were not considered in this study by the planning algorithm.

A home load control (HLC) system was put forward by the authors [22] to manage the operational planning of home appliances. The novel day-ahead HLC program was established to plan the home appliances and a plug-in hybrid electric vehicle (PHEV) in such a way as to minimize overall costs for the following day. In this work, only seven appliances, which included a heating system and a PHEV, were evaluated. Moreover, no details of the bidirectional communication between consumer and the utility were given nor information concerning the control of the residential devices by the HLC. In addition, according to the computational simulations, the use of different classes of appliances simultaneously were not considered in the new planning system proposed in this work.

A Home Energy Management as a Service (HEMaaS) method was investigated by the authors in [30]. The aim of this method was to reduce the demand at peak times and total energy consumption by moving and reducing the usage of residential appliances. HEM was expressed as a set of discrete states, which represent the binary formulation of the power levels of domestic devices. The Main Command and Control Unit (MCCU) control the power states, which were expressed as a Markov Decision Process (MDP). Reinforcement learning (RL) based on a Neural Fitted Q-Iteration (NFQI) algorithm was applied to obtain the solutions. However, the computational simulations showed that the simultaneous use of the different classes of home devices were not considered in this work when faced with the new planning criteria.

In Ref. [31], the authors proposed an algorithm that determines the thermostat settings that minimize the electricity bill for a consumer. The main goal was to use energy storage to minimize the electricity costs. The problem was formulated as dynamic programming and the results showed that the algorithm was able to reduce the cost associated with the consumption. However, it was restricted to only programming thermal devices without taking into account the other categories of home appliances. The goal of the authors in [32] was to introduce voltage hopper technology for autonomous and automated grid ancillary services and load control without a centralized controller using an electronic interface and a hybrid direct current (DC)/alternating current (AC) grid concept. Validation of the system was carried out in an interfaced dSPACE/OPAL-RT real-time simulator (Isfahan, Iran). However, the simultaneous use of the different classes of devices and the customer satisfaction were not considered.

A hybrid scheme for planning residential loads, named GAPSO, was introduced by the authors in [33]. The goal was to cut electricity cost and user discomfort but also consider the peak energy consumption. A multiple knapsack problem (MKP) was used to express the binary optimization problem. The simulation showed that GAPSO performed well to reduce costs and consumer discomfort, but the different classes of home appliances were not considered. Here, the authors in [34] developed an algorithm for the planning of residential loads to control the operational times and consumption of all the household devices. A mixed integer nonlinear programming (MINLP) problem was developed. A Benders decomposition approach was used to overcome the problem with low computational complexity. However, this work only evaluated one residence and the impact of changing the programing of the appliances was not assessed in terms of consumer satisfaction.

The authors in [35] proposed a real-time closed-loop residential electricity price-based DR system to modify consumer behavior on a smart grid. The proposal was expressed mathematically as a linear programming problem. However, neither the different classes of devices nor consumer satisfaction were evaluated. A home energy management planner algorithm to reduce residential consumption and costs using stochastic dynamic programming was presented by the authors in [36]. However, only seven home appliances were appraised and the impact of changing the times of these appliances on the consumer satisfaction was not considered.

The coordination of residential loads using a DR management distribution algorithm was presented by the authors in [37] and was expressed mathematically as a bi-level programming problem. The distributed algorithm enhanced the general load profile, the magnitude of the network voltage and the system reliability. Simulations were performed in the MATLAB environment and problems associated with home load management (HLMs’) rescheduling were solved by the General Algebraic Modeling System (GAMS). However, the simultaneous use of different classes of home devices was not assessed nor was client satisfaction evaluated.

In order to reduce the peak-to-average ratio (PAR) in aggregate load demand, two interactive algorithms based on the stochastic approximation technique were introduced by the authors in [38]. However, the algorithms did not consider the simultaneous use of different classes of residential appliances nor the client satisfaction level with this new improved planning. A DR algorithm was set up to manage energy consumption, which was expressed mathematically as a mixed integer programming problem, in order to modify residential electricity consumption profiles by the authors in [39]. The daily price of electricity and the client preferences for the use of their home appliances were taken into account, then modeled with MATLAB and solved using a GUROBI-MATLAB interface. However, only five consumers with similar consumption profiles and seven appliances were evaluated.

In [40], the authors suggested an operational planning algorithm for home appliances to reduce electricity costs based on real-time pricing. A stochastic scheduling technique based on deterministic linear programming was used to manage the times the appliances were in use. However, the different classes of the home appliances were not taken into account. A novel Traversal-and-Pruning (TP) algorithm to schedule thermostatically controlled household loads was introduced by the authors in [41]. To meet the objective of the project, both payment and comfort settings were considered. The planning of the loads was considered a mixed integer nonlinear programming (MINLP) problem. However, only thermal devices were evaluated and the other classes of domestic appliances were ignored.

Most of the recent studies presented here show that the main goal is to minimize the cost associated with the consumption of electric energy without considering the preferences/needs of end consumers. Therefore, we can say that these works do not consider the real difficulty of the problem which involves scheduling the use of home appliances and they do not evaluate aspects such as: (a) different residential scenarios; (b) various categories of home appliances; (c) the level of satisfaction/comfort of consumers with the new scheduling of their home appliances. Moreover, the studies that dealt with the inconvenience aspect performed simulations without taking into account the different categories of home appliances, thus reducing the complexity of the method.

This paper and other works in the literature have the following differences:

(1) HEMS using the EMC with the DR multi-objective optimization model allows the different categories of home appliances and the levels of satisfaction/comfort of end consumers for the new scheduling of the home appliances to be considered;
(2) The impact of different energy consumption profiles can be evaluated in relation to the management of home appliances;
(3) The HEMS using the multi-objective DR optimization model in the EMC reduced the cost of electricity for all the used scenarios, minimally affecting the satisfaction/comfort of end consumers as well as taking into account all the restrictions;
(4) HEMS can be used in any country worldwide and with any energy scenario.

## 3. Architecture of Home Energy Management System (HEMS)

HEMS is defined as the system that provides power management services in order to efficiently monitor the generation, storage and consumption of electricity in smart homes. Therefore, HEMS consists of demand response programs, automation services, power management, data visualization/analysis, auditing and security services [42].

Thus, HEMS provides a bidirectional communication between homes and the electric utility to monitor, control and analyze the data that involves the consumption of electricity in smart homes [42]. In this sense, the communication technologies, Wide Area Network (WAN), Neighborhood Area Network (NAN) and Home Area Network (HAN) [43,44,45] used in the smart grid serve as the basis for the HEMS as proposed in this work.

**Figure 1:**
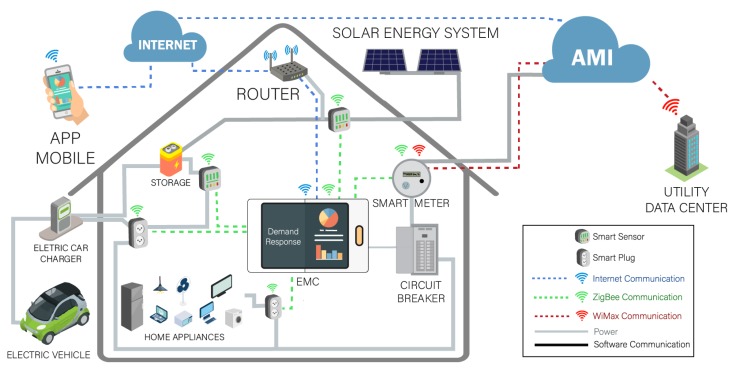
Illustration of home energy management system (HEMS) architecture.

Thus, the HEMS proposed in this work is basically composed of advanced metering infrastructure (AMI), smart meter (SM), an energy management controller (EMC) and home appliances. The architecture from HEMS is presented in Figure 1.

The smart meter is equivalent to a communication interface and is usually mounted between the AMI and EMC in order to collect the electrical energy consumption data from each device using ZigBee (IEEE 802.15.4) technology [46] and it also receives the price of electricity from the utility company in real time.

The AMI provides intelligent bidirectional communication between the SMs and the utility company. This enables automated measurement functions and also enables the utility company to send real-time data on energy consumption and price. The information is transmitted or received from the utility company through commonly available fixed networks such as PLC (Power Line Communication), GSM (Global System for Mobile Communications) or WiMax [47,48]. Thus, this data can be used for further analysis such as: each consumer’s demand for energy in a specific area or the schedules with the lowest electricity prices that can be used for moving loads.

The EMC is considered the operating nucleus of the home network and is responsible for the management of the consumption and production of energy. Based on this, the proposed HEMS can manage various devices such as electric vehicles, electrical energy storage systems, renewable energy generation, and home appliances. The HEMS uses an algorithm to allow consumers to monitor and/or reschedule the configurations of the existing devices in the residence according to their needs and the DRP data provided by the AMI, received via the smart meter.

The integration of multiple technologies combined with the optimized control of the EMC enables intelligent decision making, reliability and security. An application of this architecture envisages that the generated and stored electricity can be used over a time horizon to charge not only electric vehicles but also to provide loads to the other residential devices when, for example, the cost of electricity is high. In addition, HEMS’s communications infrastructure allows the consumers to participate actively. This is because consumers can access the whole process of monitoring, controlling and managing household energy through an Internet Mobile App. Consumers, with an HEMS Mobile App, can obtain information about energy consumption, demand and price of electricity for a certain interval of time via the SMs. Thus, consumers can make the decision to intervene or not in the optimized programming as suggested by the EMC.

**Figure 2:**
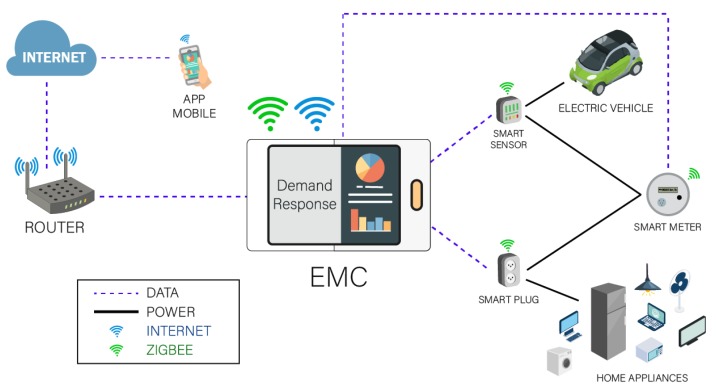
Model of an EMC communication system.

This work proposes an EMC that aims to minimize the cost associated with the consumption of electricity, the peak-to-average ratio and the level of inconvenience (dissatisfaction/discomfort) of consumers as well as to guarantee the stability and safety of the EPS. Figure 2 shows the communication between the EMC and the different devices used in the residential load management process.

In an HEMS, EMC has an important role because it manages all home appliances through the multi-objective DR model of this work and the ZigBee communication technology involved in switching gadgets on/off. The EMC schedules all operations based on the energy consumption records, the real-time electricity price and the client’s preferences. In this work, the residential devices are divided into three classes [49] as follows: interruptible and deferrable; uninterruptible and deferrable; and, uninterruptible and non-deferrable. Uninterruptible indicates that an operation cannot be interrupted until it has finished. Non-deferrable and Deferrable refer to whether an operation may start at the first time slot of the operational window, or not.

HEMS makes it easier to control and manage home appliances, to reduce the electricity consumption costs, the level of inconvenience associated with the use of appliances and it results in a lower peak-to-average ratio, which contributes to improving the reliability of the EPS operation.

## 4. Multi-Objective DR Optimization Model for Electricity Load Scheduling with NSGA-II

This section presents the multi-objective DR optimization model that will be solved using the NSGA-II algorithm to manage the loads of all the appliances taking into account the real-time pricing (RTP) structure and the operational characteristics of each appliance.

### 4.1. Mathematical Formulation

The multi-objective DR optimization model used in this work has two minimization functions: f1 and f2. The first one (*f*1) aims to minimize the electricity consumption costs and the second (*f*2) to minimize the level of inconvenience of end consumers in relation to the optimized planning of the use of residential loads provided by the utility.

The function *f*1 used in the proposed HEMS, is formulated as follows:

(1) $$\begin{array}{c} Minimize\sum_{i=1}^{N}E_{i}\sum_{t=1}^{T}{\left(Pr_{t}\cdot DSA_{t,i}\right)}^{2}, \end{array}$$

where *N* is the number of home devices; $E_{i}\left(i=1,\ldots ,N\right)$ represents the vector for the energy consumption of home devices *i* when in operation; *T* is the time horizon; $Pr_{t}$ is to the price of electric at time *t*; $DSA_{t,i}$ (Daily Setup of Appliances) refers to the load planning matrix with the following configuration:

$$DSA_{t,i}=\left\{\begin{array}{cc} 1, & \mathrm{if}\;\mathrm{appliance}\;i\;\mathrm{is}\;\mathrm{on}\;\mathrm{at}\;\mathrm{time}\;t,\\ 0, & \mathrm{otherwise}. \end{array}\right.$$

The function *f*2 aims to minimize the inconvenience and evaluate how the optimized scheduling of home appliances can modify the satisfaction/comfort of the final consumer and is given by

(2) $$\begin{array}{c} Minimize\sum_{t=1}^{T}\sum_{i=1}^{N}{\left(Baseline_{t,i}-OPT_{t,i}\right)}^{2}. \end{array}$$

Accordingly, the *f*2 calculation compares the real electricity consumption (Baseline) in the time interval *t* for the home appliance *i* of the family analyzed by the Load Profile Generator (LPG) tool [50] and the OPT consumption, which is the consumption suggested by the optimization technique, and which was used in the computational simulations. Thus, the $Baseline_{t,i}$ matrix can be defined as follows:

$$Baseline_{t,i}=\left\{\begin{array}{cc} 1, & \mathrm{if}\;\mathrm{appliance}\;i\;\mathrm{is}\;\mathrm{on}\;\mathrm{at}\;\mathrm{time}\;t,\\ 0, & \mathrm{otherwise}. \end{array}\right.$$

The $OPT_{t,i}$ consumption arises from the loading schedule suggested by the optimization technique for the various DR models defined as follows:

$$OPT_{t,i}=\left\{\begin{array}{cc} 1, & \mathrm{if}\;\mathrm{appliance}\;i\;\mathrm{is}\;\mathrm{on}\;\mathrm{at}\;\mathrm{time}\;t,\\ 0, & \mathrm{otherwise}. \end{array}\right.$$

Thus, the calculation of the inconvenience associated with optimized programming for the home appliances allows the final consumer to make the best decision on when and how to use an appliance in the DR program. Function *f*2 is shown in Equation (2) and both $Baseline_{t,i}$ and $OPT_{t,i}$ are assumed to be in the form of a binary matrix (composed only of 1’s and 0’s) to indicate which home appliances are in operation at each time interval *t*.

Equation (2) assesses the difference between the real ($Baseline_{t,i}$) and suggested consumption ($OPT_{t,i}$) for each time interval *t*, for each domestic device *i* in the problem. Thus, it compares the suggested consumption according to the proposed technique and the actual consumption of the family under analysis. Therefore, the best solution is the one where the domestic devices are only minimally affected and at the same time reduce the final cost. The closer the normal consumption is to the suggested one, the better the solution will be.

The functions *f*1 and *f*2 are subject to the following restrictions:

Constraint 1 (Equation (3)) establishes the limits (minimum and maximum) for the load levels at each time interval *t*:

(3) $$\begin{array}{c} d_{t}^{min}\leq \sum_{i=1}^{N}DSA_{t,i}\cdot P_{i}\leq d_{t}^{max},\forall_{t=1,\ldots ,T}, \end{array}$$

where $d_{t}^{min}$ is the minimum demand for the load levels at each time interval *t*; $P_{i}\left(i=1,\ldots ,N\right)$ is the vector with the power (in kW) of each home appliance; $d_{t}^{max}$ is the maximum demand for the load levels at each time interval *t*.

Constraint 2 (Equation (4)) defines the minimum ramp limit for the time interval *t*:

(4) $$\sum_{i=1}^{N}\left(DSA_{t,i}-DSA_{t+1,i}\right)\cdot P_{i}\leq r^{D},\forall_{t=1,\ldots ,T-1},$$

where $r^{D}$ is the minimum ramp limit for the time interval *t*.

Constraint 3 (Equation (5)) sets the maximum ramp limit for the time interval *t*:

(5) $$\sum_{i=1}^{N}\left(DSA_{t+1,i}-DSA_{t,i}\right)\cdot P_{i}\leq r^{U},\forall_{t=1,\ldots ,T-1},$$

where $r^{U}$ is the maximum ramp limit for the time interval *t*.

Constraint 4 (Equation (6)) defines the minimum daily consumption (mdc):

(6) $$\begin{array}{c} \sum_{i=1}^{N}\sum_{t=1}^{T}DSA_{t,i}\cdot E_{i}\geq mdc. \end{array}$$

The constraints 1–4 (Equations (3)–(6)) refer to characteristics common to power consumption. In this work, the home appliances are divided into three classes based on their operational characteristics [49] which are: interruptible and deferrable ($A_{I}$); uninterruptible and deferrable ($A_{II}$); and, uninterruptible and non-deferrable ($A_{III}$). Uninterruptible refers to an operation that cannot be interrupted until completed. Non-deferrable and Deferrable state whether the operation can begin at the first time slot of the operational window, or not. The limitations that deal with the different classes of home appliances $A_{I}$, $A_{II}$ and $A_{III}$ are based on these definitions and are specified below.

Constraint 5 (Equation (7)) states that the operational startup of category $A_{I}$ home appliances may vary over the time horizon *T* provided that $Req_{i}$ is respected:

(7) $$\begin{array}{c} \sum_{t=1}^{T}DSA_{t,i}\geq Req_{i},\forall_{i}\in A_{I}, \end{array}$$

where $Req_{i}$ is the required time for appliance *i* to finish its operation; $A_{I}$ is a set of indices of the device categories interruptible and deferrable.

Constraint 6 (Equation (8)) states that the operational startup of category $A_{II}$ home appliance can be delayed within the time horizon *T*, but, once it has started, it cannot be interrupted:

(8) $$\begin{array}{c} \sum_{q=1}^{T-(Req_{i}-1)}\prod_{t=q}^{Req_{i}+\left(q-1\right)}DSA_{t,i}\geq 1,\forall_{i}\in A_{II}, \end{array}$$

where *q* is initial time slot of the interval that will be checked if the category $A_{II}$ home appliances was used without; $A_{II}$ is a set of indices of the device categories uninterruptible and deferrable.

Constraint 7 (Equation (9)) establishes that the operation of a category $A_{III}$ home appliance between its startup ($ST_{i}$) and end ($ET_{i}$), as defined by the consumer, is uninterruptible for the required time $Req_{i}$ in the time horizon *T*:

(9) $$\begin{array}{c} \sum_{ST_{i}}^{ET_{i}}DSA_{t,i}\geq Req_{i},\forall_{i}\in A_{III}, \end{array}$$

where $ST_{i}$ is start time of the operation; $ET_{i}$ is final time of the operation; $A_{III}$ is a set of indices of the device categories uninterruptible and non-deferrable.

## 5. Case Study

This section covers the scenarios of the experiments, the results and the discussions to evaluate the HEMS using the multi-objective DR optimization algorithm to solve the scheduling problems for home appliances. In the following results, NSGA-II represents the scheduling algorithm presented.

### 5.1. Characterization of the Case Study

Three (03) different scenarios of electric power consumption were used for the simulations (Scenario 1—two adults without children; Scenario 2—two adults with three children and Scenario 3—one adult without children). These profiles were provided by the LPG [50] tool for 15 Brazilian families living in the cities of Belém—PA, Teresina—PI, Cuiabá—MT, Florianópolis—SC and São Paulo—SP, with one family per city, located in the north, northeast, midwest, south and southeast regions of Brazil, respectively. In addition, the scenario had different numbers of home appliances: Scenario 1 (29 appliances), Scenario 2 (33 appliances) and Scenario 3 (23 appliances), totaling 425 appliances for analysis. Table 1 shows the load profiles and the different categories of the home appliances.

**Figure 3:**
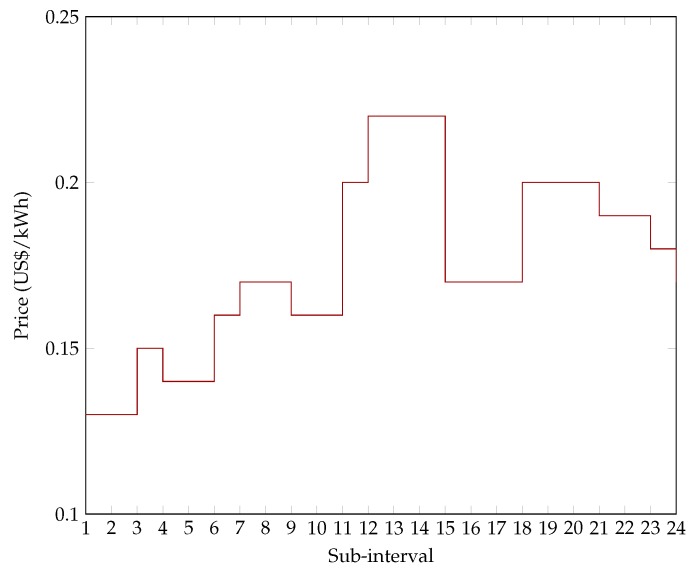
Price per unit power consumption.

The total time horizon in this study is given as *T* = 24 h. Each time interval *t* means one hour and *t* ϵ *T* such that: *T* = {1 h, 2 h …24 h}, for each family between 1 January and 31 December 2016. Other data used in the evaluations were the dynamic price of electricity in American dollars (Brazil does not use a DR program based on real-time prices). The multi-objective DR model used here allows electricity prices from studies that use forecasts or price history values to be used. Price information is an input parameter, and so the model is not restricted to the prices of any specific country or location. In such cases, RTP is considered to be the incorporated tariff. Figure 3 was created by the authors to show the price per unit power consumption at each sub-interval for an energy-intensive day (24 December 2016) of Profile I in Palmas—TO.

The parameters in NSGA-II in Table 2 were used by the DR model for validation purposes. The values of the parameter were obtained via simulations with a control map, which is a series of tests with different configurations applied to the NSGA-II. The best configuration to overcome the multi-objective problem is indicated by the NSGA-II. Other parameters such as Maximum ($d^{max}$) and Minimum Demand ($d^{min}$), Maximum ($r^{D}$) and Minimum Ramp Limit ($r^{U}$) were used with values of 3 kW, 0 kW, 1 kWh and 1 kWh, respectively.

The values first used for these parameters were based on a definition required by the consumers and the utility. Brazil has different and distinct climatic characteristics; for example, in the south and southeast regions, certain periods of the year have relatively low temperatures and therefore air conditioners are not used with much frequency; on the other hand, the north and northeast of the country are subtropical and the climate is divided into dry and rainy periods but with high temperatures all year round. Consequently, air conditioners are used much more frequently. Each city has its own distinct *mdc* value due to the different locations within Brazil and the families in this study have different energy consumption profiles. Consequently, these differences affect the final power consumption of each family differently.

### 5.2. Simulation Results

**Figure 4:**
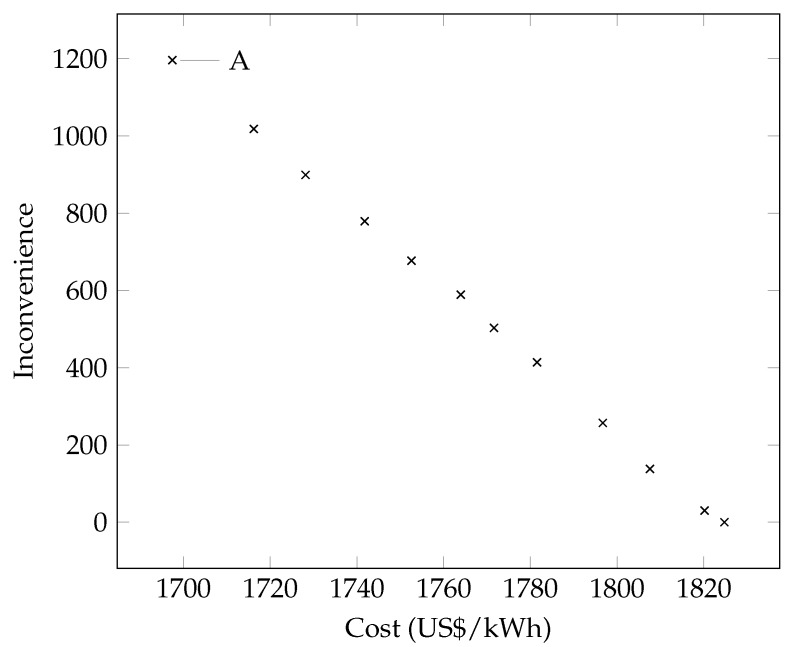
Optimal Pareto frontier.

This section presents the results associated with the scheduling of home appliances for the different scenarios of energy consumption taking into account a varied set of constraints. Thus, the impact of HEMS using the multi-objective DR optimization model was demonstrated in three aspects: (1) the cost of electricity; (2) the consumption of electricity and (3) the level of satisfaction/comfort of end consumers. In the following is a breakdown of the results for these three different scenarios. The analysis showed that the best solution is the cost minimization objective (f1, defined by Equation (1)), indicated by the letter ***A*** in Figure 4, which presents the optimal Pareto frontier reached with the experiments.

### 5.3. Scenario 1

Table 3 presents the results obtained for the cost of electric energy consumption for each family, taking into account the usage profiles of their home appliances. The results were acquired using LPG (Cost Without DR (US$)) and the technique of optimization (Cost With DR (US$)) using the DR model presented in this work. The table shows that the family living in Teresina—PI compared to the other families in the other cities (Belém—PA, Cuiabá—MT, Florianópolis—SC and São Paulo—SP) obtained the largest cost reduction: dropping from US$ 99.31 to US$ 90.72.

Another analysis evaluated the HEMS using the DR optimization model for the reduction of the consumption. Table 4 shows that the family residing in Teresina—PI reduced their consumption from 1891.45 kWh to 1709.57 kWh.

The power consumption of domestic devices is compared on an hourly basis in kWh, with DR (driven by HEMS) and without DR (base consumption) in Table 5. This DR model moves the operational times to when the electricity price is cheaper (off-peak). For example, the electric stove of the family in Teresina—PI, runs without DR between 2:00 p.m. and 5:00 p.m., and with DR its operation is transferred to between 4:00 p.m. and 7:00 p.m. The model with DR demonstrated a good reduction in the total cost: without DR US$ 0.20, with DR US$ 0.17, making a savings of US$ 0.03.

Based on the results obtained, the *trade-off* solution was calculated, that is, the relation between each unit of inconvenience caused to an end consumer and the reduction attributed to it, which results in the total reduction (in US$) obtained with each unit of inconvenience caused. Thus, the highest *trade-off* value found was 0.11 for the family located in Teresina—PI, which is equivalent to a reduction of US$ 0.11 per unit of inconvenience caused to the end consumer. Table 6 shows a summary of the results for the inconvenience and *trade-off* simulations for each family resident in Belém—PA, Cuiabá—MT, Florianópolis—SC, São Paulo—SP and Teresina—PI.

### 5.4. Scenario 2

The results show that the family living in Teresina—PI reduced the total electricity cost from US$ 250.66 to US$ 229.08. Thus, the family living in Teresina—PI had the highest values related to cost minimization associated with the consumption of electric energy compared to other families in the cities of Belém—PA, Cuiabá—MT, Florianópolis—SC and São Paulo—SP. Table 7 shows a summary of the results achieved for Scenario 2.

Table 8 shows a comparison of the electric energy consumption of each family in each city. Once again, the family residing in Teresina—PI obtained the greatest reduction in consumption, dropping from 4774.09 kWh to 4316.96 kWh.

Table 9 is a comparison of the consumption (in kWh) for each hour of the domestic devices considering the base consumption without DR and with DR driven by HEMS using the model proposed here. For example, the electric stove of the family in Cuiabá—MT, runs without DR between 2:00 p.m. and 5:00 p.m. and with DR its operation is moved to between 4:00 p.m. and 7:00 p.m. The HEMS compared with the base consumption gives a satisfactory reduction in the total cost: without DR US$ 0.69, with DR US$ 0.52, giving a savings of US$ 0.17.

The inconvenience and *trade-off* analysis shows that the family resident in Teresina—PI obtained the highest *trade-off* value with a total of 0.17, which is equivalent to US$ 0.17 reduction per unit of inconvenience caused to the end consumer. Table 10 shows a summary of the results obtained in the study for the inconvenience and the *trade-off* for the families in their respective cities.

### 5.5. Scenario 3

Table 11 gives a summary of the electricity cost for each family in Scenario 3. The family residing in Teresina—PI managed to obtain a greater reduction compared to the other families, with the cost dropping from US$ 57.45 to US$ 52.52.

Table 12 shows a comparison of the electric energy consumption of each family in each city. The simulation results show that the family living in Teresina—PI reduced their consumption from 1094.23 kWh to 989.75 kWh.

Table 13 gives a per hour comparison of the consumption (in kWh) of the domestic devices considering consumption with DR and without DR driven by HEMS. For example, the microwave of the family in São Paulo—SP runs without DR between 2:00 p.m. and 6:00 p.m. and with DR, its operation is moved to between 3:00 p.m. and 7:00 p.m. This change with HEMS gives a good reduction in the total cost: without DR US$ 0.79, with DR US$ 0.57, giving a savings of US$ 0.22.

Table 14 summarizes the results for the inconvenience and *trade-off*. The inconvenience and *trade-off* values show that the family residing in Teresina—PI had the highest *trade-off* value which was 0.12, and this is equivalent to a US$ 0.12 reduction per unit of inconvenience caused to the end consumer.

### 5.6. Statistical Analysis

The results from the experiments with scheduling for the home appliances were analyzed by three performance metrics: Diversity, Coverage, and Hypervolume. Diversity [51] measures the number of different solutions given by an algorithm in a search space between extreme solutions (maximum/minimum solutions of each objective function). Thus, a great number of solutions found in the search space means there are a great number of options available for decision-making.

The Coverage (metric C) is used to evaluate the optimal approach capability of the solutions, which is the (theoretical) distance between the current Pareto Frontier and the theoretical optimal Pareto Frontier. Thus, based on its theoretical properties [52], coverage ensures a space of solutions closer to the theoretical optimum to solve the DR problem.

Simulations with a random search algorithm are used to calculate the Coverage metric. A random search algorithm is a genetic algorithm (GA) [53], with a random selection method that does not use heuristics, called random GA, and is compared to the NSGA-II optimization technique. Therefore, the C [52] metric is used to determine which of the techniques (NSGA-II or random GA) has the best coverage. The Hypervolume (HV) metric [52,54] is used to evaluate the overall performance of the two techniques (NSGA-II or random GA) in more detail. Both NSGA-II and random GA were performed 1000 executions to reduce the impact of their stochastic nature and to obtain the values to be used in the statistical analysis.

#### 5.6.1. Diversity

The spacing metric [51] was used to calculate the Diversity, which is given by *s*:

(10) $$s=\sqrt{\frac{1}{n-1}\sum_{i=1}^{n}{\left(\bar{d}-d_{i}\right)}^{2}},$$

where $d_{i}$ = $\min_{j}\sum_{a=1}^{M}\left(|f_{a}^{i}\left(x\right)-f_{a}^{j}\left(x\right)|\right)$, *i*, *j* = 1, 2, 3,…, *n*, i≠j. $\bar{d}$ represents the average values of $d_{i}$, *M* is the number of objectives of the problem and *n* is the number of solutions.

The closer the value of *s* is to zero, the greater the similarity between the solutions will be, within the analyzed set. Thus, there will be a lower diversity of solutions [51].

#### 5.6.2. Coverage

The coverage ratio for two sets of solutions is compared by the metric *C*. The number of points in set *B* dominated by set *A* over the total number of points in set *B* is represented by C(A,B). Equation (11) demonstrates metric *C*:

(11) $$C\left(A,B\right)=\frac{|\left\{x\in B|\exists y\in A:y\;\mathrm{dominates}\;x\right\}|}{|B|}.$$

If the value C(A,B)=1, it means that all points in *B* are dominated by *A* or equal to the points contained in *A*. In contrast, if C(A,B)=0, it indicates that none of the points in *B* are dominated by the set *A*. Thus, it should be noted that C(A,B) and C(B,A) should be considered because C(A,B) ≠ 1−C(B,A) [52]. In the simulations, *A* will be composed of the Pareto frontier solutions of the DR multi-objective model presented in this work using the NSGA-II, while *B* will be composed of the Pareto frontier solutions of the random search algorithm.

#### 5.6.3. Hypervolume

The Hypervolume (HV) was calculated to analyze the performance of the results from the DR model. An HV is a performance metric that calculates the volume of the objective space among the set of solutions found and a reference point; here, the reference point was the nadir point, which is the vector whose elements are the worst values of each criterion of the multi-objective problem [55,56]. The higher the HV value is, the better the convergence, extension and uniformity [52].

The authors in [54] state that HV is the only unary metric that is able to evaluate if one set of solutions *S* is not worse than another *S* set. Thus, a set of solutions is Pareto optima only when the HV is maximized and vice versa [57]. Thus, the main characteristic of HV is that it is compatible with the dominance of Pareto; if one population of Pareto dominates another, then this one has an HV greater than the dominated one. In addition, it does not need the real Pareto frontier of the problem in its calculation [52,54].

#### 5.6.4. Statistical Results

The results of the study with the NSGA-II optimization technique were compared with the values from the random GA in order to validate the correctness of the algorithm (*sanity check*). The values of the spacing metric showed that the NSGA-II (minimum 14.32 and maximum 18.11) had a greater diversity of solutions than the random GA (minimum 10.25 and maximum 15.96) Therefore, the NSGA-II had a better coverage of the search space, and this translates into a better comprehension of the objectives considered in the problem.

In the metric C, the values obtained for both C(A,B) and C(B,A) indicate that, in all cases, the Pareto frontier solutions found by the NSGA-II completely dominated the frontier solutions of Pareto found by random GA. Additionally, it utilized the time spent (milliseconds) in solving the problem as another evaluation metric. This result shows that the NSGA-II presents better solutions than the random GA, considering the Pareto frontier of both techniques.

Additionally, the analysis of the Hypervolume values found in the simulations indicates a significantly better general performance of NSGA-II (minimum 0.55 and maximum 0.63) in relation to random GA (minimum 0.34 and maximum 0.45). This information, as previously mentioned, reflects the better performance, in terms of convergence and extension, of the solution considering the search space [52]. Finally, it can be seen in the statistical results that the NSGA-II obtained a minimum execution time of 56 and a maximum of 70, while the Random GA presented a minimum execution time of 60 and a maximum of 77. Therefore, both the NSGA-II and Random GA with these execution times enable the load scheduling to provide a reduction of electricity costs, as well as minimize the inconvenience caused to the end consumers in a timely manner. Table 15 shows the statistical values for the simulations.

## 6. Conclusions

Scheduling management of home appliances in smart grids enables the EPS to be more efficient and effective because issues such as power interruptions during peak demands can be minimized. Thus, DR plays a key role in managing energy consumption in order to avoid overloading as well as reducing the cost of electricity for end consumers. However, this optimized operation of home appliances requires an infrastructure capable of scheduling the operating periods of the devices over the planning horizon, and thus reducing the periods of peak demand, and improving the reliability and efficiency of the EPS minimally affecting the satisfaction/comfort of end consumers. This paper proposes an architecture of a home energy management system (HEMS) and presents a multi-objective DR optimization model to manage the scheduling of electrical appliances in residencies, aiming at minimizing the cost associated to the energy consumption, as well as minimizing the inconvenience (dissatisfaction/discomfort) of end consumers.

The performance of the HEMS using the DR optimization model was evaluated through simulations. First, the efficiency of the HEMS was analyzed for cost minimization associated with the consumption of electric energy as well as inconvenience (dissatisfaction/discomfort) minimization of end consumers of the different residential Scenarios. In addition, the HEMS performance was evaluated for the load scheduling of some appliances (Table 5, Table 9 and Table 13) in order to verify the influence of such appliances to reduce the cost of electricity. Next, through the diversity, coverage and hypervolume metrics, the characteristics of the solutions for the problem of scheduling the home appliances were evaluated.

The results of the study showed that there is a significant reduction in the total cost associated with the consumption of electric power for the three scenarios analyzed. The families that obtained the greatest reductions were the residents in Teresina—PI where the total cost of electricity was reduced from US$ 99.31 to US$ 90.72, from US$ 250.66 to US$ 229.08, and from US$ 57.45 to US$ 52.52 for Scenarios 1, 2 and 3, respectively. Moreover, when the level of inconvenience and the trade-off were analyzed, after optimizing the use of home appliances, the highest values for *trade-off* were 0.11, 0.17 and 0.12 for the Scenarios 1, 2 and 3, respectively, for the families living in Teresina—PI. Thus, these families achieved a reduction in the price of electricity equivalent to US$ 0.11, US$ 0.17 and US$ 0.12. In addition, the statistical results in Table 15 show that, when the HEMS applied the NSGA-II technique, with the multi-objective DR model, in the EMC, it obtained the best results of the simulations when compared to the random search algorithm for all the metrics (Diversity, Coverage and Hypervolume) used in this work.

Future research could further improve in several directions. One possibility would be to improve the model so that some microgrid characteristics, such as the use of electric vehicles and renewable sources for the generation of electric energy, can be included. Another direction could be to evaluate the performance of our model using the NSGA-II technique for other residential scenarios. In addition, a third option could be to solve the multi-objective problems presented in this work with other optimization techniques.

## Figures and Tables

**Table 1:** Load profiles and categories of home appliances.

Profiles	Categories	Home Appliances
**Profile I**	$A_{I}$	Light 100 W, 20 W and 60 W, SAT—Receiver, TV, Cell Phone Charging, Microsoft Xbox, Laptop, CD/DVD Player, Computer, DVB—T Receiver, Router, Computer Screen, Kitchen Radio.
	$A_{II}$	Wine Cellar, Steam Iron, Hair Dryer, Electric Stove, Microwave, Juicer, Washing Machine, Toaster, Electric Kettle, Nespresso Coffee Machine.
	$A_{III}$	Refrigerator, Air Conditioning, Electric Heater, Freezer, Dryer.
**Profile II**	$A_{I}$	Light 100 W, 20 W and 60 W, SAT—Receiver, TV, Cell Phone Charging, Playstation, Microsoft Xbox, Laptop, CD/DVD Player, Computer, Home Cinema System, DVB—T Receiver, Router, Computer Screen, Kitchen Radio.
	$A_{II}$	Wine Cellar, Steam Iron, Hair Dryer, Electric Razor, Electric Stove, Electronic Hometrainer, Microwave, Juicer, Washing Machine, Toaster, Electric Kettle, Nespresso Coffee Machine.
	$A_{III}$	Refrigerator, Air Conditioning, Electric Heater, Freezer, Dryer.
**Profile III**	$A_{I}$	Light 100 W, 20 W and 60 W, SAT—Receiver, TV, Playstation, Laptop, CD/DVD Player, Computer, DVB—T Receiver, Router, Computer Screen.
	$A_{II}$	Wine Cellar, Steam Iron, Food Multiprocessor, Microwave, Washing Machine, Electric Kettle, Nespresso Coffee Machine.
	$A_{III}$	Refrigerator, Air Conditioning, Electric Heater, Freezer.

**Table 2:** NSGA-II parameters.

Parameter	Value
Population size	500
Maximum number of iterations	1.000
Selection method	Tournament (3)
Crossover method	Single Point
Crossover probability	85%
Mutation method	Bit Flip
Mutation probability	1%

**Table 3:** Reduction of electricity costs per family in Scenario 1 for each city.

Family	City	Without DR (US$)	With DR (US$)	Reduction (%)	Reduction (US$)
I	Belém—PA	92.09	87.42	5.06	4.66
II	Cuiabá—MT	97.78	90.48	7.46	7.29
III	Florianópolis—SC	84.45	78.48	7.07	5.97
IV	São Paulo—SP	88.96	83.35	6.31	5.61
V	Teresina—PI	99.31	90.72	8.65	8.59

**Table 4:** Reduction of energy consumption per family in Scenario 1 for each city.

Family	City	Without DR (kWh)	With DR (kWh)	Reduction (%)	Reduction (kWh)
I	Belém—PA	1684.17	1597.64	5.14	86.53
II	Cuiabá—MT	1937.84	1771.05	8.61	166.79
III	Florianópolis—SC	1737.39	1685.25	3.00	52.13
IV	São Paulo—SP	1637.76	1550.90	5.30	86.86
V	Teresina—PI	1891.45	1709.57	9.62	181.87

**Table 5:** Comparison of the electric energy consumption of home appliance with and without optimization (in kWh) in Scenario 1.

Cities	Home Appliances	DR	01:00	02:00	03:00	04:00	05:00	06:00	07:00	08:00	09:00	10:00	11:00	12:00	13:00	14:00	15:00	16:00	17:00	18:00	19:00	20:00	21:00	22:00	23:00	24:00	Total Cost (US$)
Cuiabá—MT	Stove	Without	0	0	0	0	0	0	0	0	0	0	0	3	3	3	3	0	0	0	0	0	0	0	0	0	0.95
		With	0	0	3	3	3	3	0	0	0	0	0	0	0	0	0	0	0	0	0	0	0	0	0	0	0.67
	Computer	Without	0.3	0	0	0	0.3	0.3	0.3	0	0	0	0	0	0	0	0	0	0.3	0.3	0	0.3	0	0	0	0.3	0.16
		With	0.3	0.3	0.3	0.3	0.3	0.3	0.3	0	0	0	0	0	0	0	0	0	0	0	0	0	0	0	0	0.3	0.14
	Washing Machine	Without	0	0	0	0	1	1	1	1	1	0	0	0	0	0	0	0	0	0	0	0	0	0	0	0	0.32
		With	0	1	1	1	1	1	0	0	0	0	0	0	0	0	0	0	0	0	0	0	0	0	0	0	0.28
	Oven	Without	0	0	0	0	0	0	0	0	0	0	0	1.5	1.5	1.5	1.5	0	0	0	0	0	0	0	0	0	0.47
		With	0	0	1.5	1.5	1.5	1.5	0	0	0	0	0	0	0	0	0	0	0	0	0	0	0	0	0	0	0.34
	Microwave	Without	0	0	0	0	0	0	0	0	0	2	2	2	2	2	0	0	0	0	0	0	0	0	0	0	0.79
		With	0	2	2	2	2	2	0	0	0	0	0	0	0	0	0	0	0	0	0	0	0	0	0	0	0.57
	Energy Price (US$/kWh)		0.06	0.06	0.05	0.06	0.05	0.06	0.06	0.07	0.08	0.08	0.08	0.08	0.08	0.08	0.08	0.08	0.08	0.08	0.08	0.07	0.07	0.08	0.07	0.06	–
São Paulo—SP	Stove	Without	0	0	0	0	0	0	0	0	0	0	0	3	3	3	3	0	0	0	0	0	0	0	0	0	0.69
		With	0	0	0	3	3	3	3	0	0	0	0	0	0	0	0	0	0	0	0	0	0	0	0	0	0.52
	Computer	Without	0	0	0	0	0.3	0.3	0.3	0.3	0.3	0	0	0	0	0	0	0	0.3	0.3	0	0.3	0	0	0	0	0.12
		With	0	0.3	0.3	0.3	0.3	0.3	0.3	0.3	0.3	0	0	0	0	0	0	0	0	0	0	0	0	0	0	0	0.11
	Washing Machine	Without	0	0	0	0	1	1	1	1	1	0	0	0	0	0	0	0	0	0	0	0	0	0	0	0	0.23
		With	0	1	1	1	1	1	0	0	0	0	0	0	0	0	0	0	0	0	0	0	0	0	0	0	0.22
	Oven	Without	0	0	0	1.5	1.5	1.5	1.5	0	0	0	0	0	0	0	0	0	0	0	0	0	0	0	0	0	0.34
		With	0	0	0	0	0	0	0	0	0	0	0	1.5	1.5	1.5	1.5	0	0	0	0	0	0	0	0	0	0.26
	Microwave	Without	0	0	0	0	0	0	0	0	0	2	2	2	2	2	0	0	0	0	0	0	0	0	0	0	0.55
		With	0	0	0	2	2	2	2	2	0	0	0	0	0	0	0	0	0	0	0	0	0	0	0	0	0.44
	Energy Price (US$/kWh)		0.05	0.05	0.05	0.04	0.04	0.04	0.04	0.05	0.05	0.05	0.05	0.06	0.06	0.06	0.06	0.05	0.05	0.06	0.07	0.07	0.08	0.08	0.07	0.06	–
Teresina—PI	Stove	Without	0	0	0	0	0	0	0	0	0	0	0	0	0	3	3	3	3	0	0	0	0	0	0	0	0.40
		With	0	0	0	0	0	0	0	0	0	0	0	0	0	0	0	3	3	3	3	0	0	0	0	0	0.34
	Computer	Without	0	0	0	0	0	0	0.3	0.3	0.3	0	0	0	0	0	0	0	0	0.3	0.3	0.3	0.3	0.3	0	0	0.09
		With	0	0	0	0	0	0.3	0.3	0.3	0	0	0	0	0	0	0	0.3	0.3	0.3	0.3	0.3	0	0	0	0	0.08
	Washing Machine	Without	0	0	0	0	1	1	1	1	1	0	0	0	0	0	0	0	0	0	0	0	0	0	0	0	0.19
		With	0	1	1	1	1	1	0	0	0	0	0	0	0	0	0	0	0	0	0	0	0	0	0	0	0.19
	Oven	Without	0	0	0	0	0	0	0	0	0	0	0	0	0	1.5	1.5	1.5	1.5	0	0	0	0	0	0	0	0.20
		With	0	0	0	0	0	0	0	0	0	0	0	0	0	0	0	1.5	1.5	1.5	1.5	0	0	0	0	0	0.17
	Microwave	Without	0	0	0	0	0	0	0	0	0	0	0	0	0	2	2	2	2	2	0	0	0	0	0	0	0.32
		With	0	0	0	0	0	0	0	0	0	0	0	0	0	0	2	2	2	2	2	0	0	0	0	0	0.30
	Energy Price (US$/kWh)		0.05	0.04	0.04	0.04	0.04	0.04	0.04	0.04	0.04	0.04	0.05	0.04	0.04	0.04	0.04	0.03	0.03	0.03	0.03	0.04	0.05	0.05	0.05	0.05	–

**Table 6:** Inconvenience analysis and *trade-off* in Scenario 1.

Family	City	Inconvenience Caused	*Trade-off*
I	Belém—PA	72	0.07
II	Cuiabá—MT	76	0.09
III	Florianópolis—SC	70	0.09
IV	São Paulo—SP	73	0.08
V	Teresina—PI	75	0.11

**Table 7:** Reduction of electricity costs per family in Scenario 2 for each city.

Family	City	Without DR (US$)	With DR (US$)	Reduction (%)	Reduction (US$)
I	Belém—PA	216.96	205.93	5.08	11.02
II	Cuiabá—MT	229.32	212.17	7.48	17.15
III	Florianópolis—SC	199.89	185.49	7.20	14.39
IV	São Paulo—SP	208.12	194.86	6.37	13.26
V	Teresina—PI	250.66	229.08	8.61	21.58

**Table 8:** Reduction of energy consumption per family in Scenario 2 for each city.

Family	City	Without DR (kWh)	With DR (kWh)	Reduction (%)	Reduction (kWh)
I	Belém—PA	3967.96	3763.35	5.16	204.62
II	Cuiabá—MT	4544.95	4152.92	8.63	392.03
III	Florianópolis—SC	4112.01	3983.02	3.14	128.99
IV	São Paulo—SP	3831.38	3625.93	5.36	205.45
V	Teresina—PI	4774.09	4316.96	9.58	457.14

**Table 9:** Comparison of the electric energy consumption of home appliances with and without optimization (in kWh) in Scenario 2.

Cities	Home Appliances	DR	01:00	02:00	03:00	04:00	05:00	06:00	07:00	08:00	09:00	10:00	11:00	12:00	13:00	14:00	15:00	16:00	17:00	18:00	19:00	20:00	21:00	22:00	23:00	24:00	Total Cost (US$)
Cuiabá—MT	Stove	Without	0	0	0	0	0	0	0	0	0	0	0	0	0	3	3	3	3	0	0	0	0	0	0	0	0.69
		With	0	0	0	0	0	0	0	0	0	0	0	0	0	0	0	3	3	3	3	0	0	0	0	0	0.52
	Computer	Without	0	0	0	0	0	0	0.3	0.3	0.3	0	0	0	0	0	0	0	0	0.3	0.3	0.3	0.3	0.3	0	0	0.12
		With	0	0	0	0	0	0.3	0.3	0.3	0	0	0	0	0	0	0	0.3	0.3	0.3	0.3	0.3	0	0	0	0	0.11
	Washing Machine	Without	0	0	0	0	1	1	1	1	1	0	0	0	0	0	0	0	0	0	0	0	0	0	0	0	0.23
		With	0	1	1	1	1	1	0	0	0	0	0	0	0	0	0	0	0	0	0	0	0	0	0	0	0.22
	Oven	Without	0	0	0	0	0	0	0	0	0	0	0	0	0	1.5	1.5	1.5	1.5	0	0	0	0	0	0	0	0.34
		With	0	0	0	0	0	0	0	0	0	0	0	0	0	0	0	1.5	1.5	1.5	1.5	0	0	0	0	0	0.26
	Microwave	Without	0	0	0	0	0	0	0	0	0	0	0	0	0	2	2	2	2	2	0	0	0	0	0	0	0.55
		With	0	0	0	0	0	0	0	0	0	0	0	0	0	0	2	2	2	2	2	0	0	0	0	0	0.44
	Energy Price (US$/kWh)		0.05	0.04	0.04	0.04	0.04	0.04	0.04	0.04	0.04	0.04	0.05	0.04	0.04	0.04	0.04	0.03	0.03	0.03	0.03	0.04	0.05	0.05	0.05	0.05	–
São Paulo—SP	Stove	Without	0	0	0	0	0	0	0	0	0	0	0	3	3	3	3	0	0	0	0	0	0	0	0	0	0.40
		With	0	0	0	3	3	3	3	0	0	0	0	0	0	0	0	0	0	0	0	0	0	0	0	0	0.34
	Computer	Without	0	0	0	0	0.3	0.3	0.3	0.3	0.3	0	0	0	0	0	0	0	0.3	0.3	0	0.3	0	0	0	0	0.09
		With	0	0.3	0.3	0.3	0.3	0.3	0.3	0.3	0.3	0	0	0	0	0	0	0	0	0	0	0	0	0	0	0	0.08
	Washing Machine	Without	0	0	0	0	1	1	1	1	1	0	0	0	0	0	0	0	0	0	0	0	0	0	0	0	0.19
		With	0	1	1	1	1	1	0	0	0	0	0	0	0	0	0	0	0	0	0	0	0	0	0	0	0.19
	Oven	Without	0	0	0	1.5	1.5	1.5	1.5	0	0	0	0	0	0	0	0	0	0	0	0	0	0	0	0	0	0.20
		With	0	0	0	0	0	0	0	0	0	0	0	1.5	1.5	1.5	1.5	0	0	0	0	0	0	0	0	0	0.17
	Microwave	Without	0	0	0	0	0	0	0	0	0	2	2	2	2	2	0	0	0	0	0	0	0	0	0	0	0.32
		With	0	0	0	2	2	2	2	2	0	0	0	0	0	0	0	0	0	0	0	0	0	0	0	0	0.30
	Energy Price (US$/kWh)		0.05	0.05	0.05	0.05	0.05	0.05	0.05	0.05	0.05	0.05	0.05	0.06	0.06	0.06	0.06	0.05	0.05	0.06	0.07	0.07	0.07	0.07	0.07	0.06	–
Teresina—PI	Stove	Without	0	0	0	0	0	0	0	0	0	0	0	3	3	3	3	0	0	0	0	0	0	0	0	0	0.95
		With	0	0	3	3	3	3	0	0	0	0	0	0	0	0	0	0	0	0	0	0	0	0	0	0	0.67
	Computer	Without	0.3	0	0	0	0.3	0.3	0.3	0	0	0	0	0	0	0	0	0	0.3	0.3	0	0.3	0	0	0	0.3	0.16
		With	0.3	0.3	0.3	0.3	0.3	0.3	0.3	0	0	0	0	0	0	0	0	0	0	0	0	0	0	0	0	0.3	0.14
	Washing Machine	Without	0	0	0	0	1	1	1	1	1	0	0	0	0	0	0	0	0	0	0	0	0	0	0	0	0.32
		With	0	1	1	1	1	1	0	0	0	0	0	0	0	0	0	0	0	0	0	0	0	0	0	0	0.28
	Oven	Without	0	0	0	0	0	0	0	0	0	0	0	1.5	1.5	1.5	1.5	0	0	0	0	0	0	0	0	0	0.47
		With	0	0	1.5	1.5	1.5	1.5	0	0	0	0	0	0	0	0	0	0	0	0	0	0	0	0	0	0	0.34
	Microwave	Without	0	0	0	0	0	0	0	0	0	2	2	2	2	2	0	0	0	0	0	0	0	0	0	0	0.79
		With	0	2	2	2	2	2	0	0	0	0	0	0	0	0	0	0	0	0	0	0	0	0	0	0	0.57
	Energy Price (US$/kWh)		0.06	0.06	0.06	0.06	0.06	0.06	0.06	0.07	0.08	0.08	0.08	0.08	0.08	0.08	0.08	0.08	0.07	0.07	0.07	0.07	0.07	0.08	0.07	0.06	–

**Table 10:** Inconvenience and *trade-off* analysis in Scenario 2.

Family	City	Inconvenience Caused	*Trade-off*
I	Belém—PA	125	0.09
I	Cuiabá—MT	127	0.12
III	Florianópolis—SC	123	0.12
IV	São Paulo—SP	124	0.11
V	Teresina—PI	126	0.17

**Table 11:** Reduction in electricity costs per family in Scenario 3 for each city.

Family	City	Without DR (US$)	With DR (US$)	Reduction (%)	Reduction (US$)
I	Belém—PA	50.44	47.88	5.07	2.56
II	Cuiabá—MT	50.34	46.61	7.42	3.74
III	Florianópolis—SC	49.06	45.59	7.07	3.47
IV	São Paulo—SP	49.95	46.86	6.19	3.09
V	Teresina—PI	57.45	52.52	8.58	4.93

**Table 12:** Reduction of electric energy consumption per family in Scenario 3 for each city.

Family	City	Without DR (kWh)	With DR (kWh)	Reduction (%)	Reduction (kWh)
I	Belém—PA	922.46	874.99	5.15	47.47
II	Cuiabá—MT	997.75	912.29	8.57	85.46
III	Florianópolis—SC	1009.22	978.94	3.00	30.28
IV	São Paulo—SP	919.52	871.92	5.18	47.60
V	Teresina—PI	1094.23	989.75	9.55	104.48

**Table 13:** Comparison of the electric energy consumption of home appliances with and without optimization (in kWh) in Scenario 3.

Cities	Home Appliances	DR	01:00	02:00	03:00	04:00	05:00	06:00	07:00	08:00	09:00	10:00	11:00	12:00	13:00	14:00	15:00	16:00	17:00	18:00	19:00	20:00	21:00	22:00	23:00	24:00	Total Cost (US$)
Cuiabá—MT	Stove	Without	0	0	0	0	0	0	0	0	0	0	0	3	3	3	3	0	0	0	0	0	0	0	0	0	0.40
		With	0	0	0	3	3	3	3	0	0	0	0	0	0	0	0	0	0	0	0	0	0	0	0	0	0.34
	Computer	Without	0	0	0	0	0.3	0.3	0.3	0.3	0.3	0	0	0	0	0	0	0	0.3	0.3	0	0.3	0	0	0	0	0.09
		With	0	0.3	0.3	0.3	0.3	0.3	0.3	0.3	0.3	0	0	0	0	0	0	0	0	0	0	0	0	0	0	0	0.08
	Washing Machine	Without	0	0	0	0	1	1	1	1	1	0	0	0	0	0	0	0	0	0	0	0	0	0	0	0	0.19
		With	0	1	1	1	1	1	0	0	0	0	0	0	0	0	0	0	0	0	0	0	0	0	0	0	0.19
	Oven	Without	0	0	0	1.5	1.5	1.5	1.5	0	0	0	0	0	0	0	0	0	0	0	0	0	0	0	0	0	0.20
		With	0	0	0	0	0	0	0	0	0	0	0	1.5	1.5	1.5	1.5	0	0	0	0	0	0	0	0	0	0.17
	Microwave	Without	0	0	0	0	0	0	0	0	0	2	2	2	2	2	0	0	0	0	0	0	0	0	0	0	0.32
		With	0	0	0	2	2	2	2	2	0	0	0	0	0	0	0	0	0	0	0	0	0	0	0	0	0.30
	Energy Price (US$/kWh)		0.05	0.05	0.05	0.04	0.04	0.04	0.05	0.05	0.05	0.05	0.05	0.06	0.06	0.06	0.06	0.05	0.05	0.06	0.07	0.07	0.07	0.07	0.07	0.06	–
São Paulo—SP	Stove	Without	0	0	0	0	0	0	0	0	0	0	0	0	0	3	3	3	3	0	0	0	0	0	0	0	0.95
		With	0	0	0	0	0	0	0	0	0	0	0	0	0	0	0	3	3	3	3	0	0	0	0	0	0.67
	Computer	Without	0	0	0	0	0	0	0.3	0.3	0.3	0	0	0	0	0	0	0	0	0.3	0.3	0.3	0.3	0.3	0	0	0.16
		With	0	0	0	0	0	0.3	0.3	0.3	0	0	0	0	0	0	0	0.3	0.3	0.3	0.3	0.3	0	0	0	0	0.14
	Washing Machine	Without	0	0	0	0	1	1	1	1	1	0	0	0	0	0	0	0	0	0	0	0	0	0	0	0	0.32
		With	0	1	1	1	1	1	0	0	0	0	0	0	0	0	0	0	0	0	0	0	0	0	0	0	0.28
	Oven	Without	0	0	0	0	0	0	0	0	0	0	0	0	0	1.5	1.5	1.5	1.5	0	0	0	0	0	0	0	0.47
		With	0	0	0	0	0	0	0	0	0	0	0	0	0	0	0	1.5	1.5	1.5	1.5	0	0	0	0	0	0.34
	Microwave	Without	0	0	0	0	0	0	0	0	0	0	0	0	0	2	2	2	2	2	0	0	0	0	0	0	0.79
		With	0	0	0	0	0	0	0	0	0	0	0	0	0	0	2	2	2	2	2	0	0	0	0	0	0.57
	Energy Price (US$/kWh)		0.05	0.04	0.04	0.04	0.04	0.04	0.04	0.04	0.04	0.04	0.05	0.04	0.04	0.04	0.04	0.03	0.03	0.03	0.03	0.04	0.05	0.05	0.05	0.05	–
Teresina—PI	Stove	Without	0	0	0	0	0	0	0	0	0	0	0	3	3	3	3	0	0	0	0	0	0	0	0	0	0.69
		With	0	0	3	3	3	3	0	0	0	0	0	0	0	0	0	0	0	0	0	0	0	0	0	0	0.52
	Computer	Without	0.3	0	0	0	0.3	0.3	0.3	0	0	0	0	0	0	0	0	0	0.3	0.3	0	0.3	0	0	0	0.3	0.12
		With	0.3	0.3	0.3	0.3	0.3	0.3	0.3	0	0	0	0	0	0	0	0	0	0	0	0	0	0	0	0	0.3	0.11
	Washing Machine	Without	0	0	0	0	1	1	1	1	1	0	0	0	0	0	0	0	0	0	0	0	0	0	0	0	0.23
		With	0	1	1	1	1	1	0	0	0	0	0	0	0	0	0	0	0	0	0	0	0	0	0	0	0.22
	Oven	Without	0	0	0	0	0	0	0	0	0	0	0	1.5	1.5	1.5	1.5	0	0	0	0	0	0	0	0	0	0.34
		With	0	0	1.5	1.5	1.5	1.5	0	0	0	0	0	0	0	0	0	0	0	0	0	0	0	0	0	0	0.26
	Microwave	Without	0	0	0	0	0	0	0	0	0	2	2	2	2	2	0	0	0	0	0	0	0	0	0	0	0.55
		With	0	2	2	2	2	2	0	0	0	0	0	0	0	0	0	0	0	0	0	0	0	0	0	0	0.44
	Energy Price (US$/kWh)		0.06	0.06	0.06	0.06	0.06	0.06	0.06	0.07	0.08	0.08	0.08	0.08	0.08	0.08	0.08	0.08	0.07	0.07	0.07	0.07	0.07	0.08	0.07	0.06	–

**Table 14:** Inconvenience and *trade-off* analysis in Scenario 3.

Family	City	Inconvenience Caused	*Trade-off*
I	Belém—PA	42	0.06
II	Cuiabá—MT	43	0.09
III	Florianópolis—SC	39	0.09
IV	São Paulo—SP	40	0.08
V	Teresina—PI	41	0.12

**Table 15:** Statistical analysis.

Algorithm	Metric	Min	Max	Average	Standard Deviation
NSGA-II	*Spacing*	14.32	18.11	16.06	1.14
Random GA		10.25	15.96	14.37	1.06
NSGA-II	C (A, B)	1	1	1	0
Random GA					
Random GA	C (B, A)	0	0	0	0
NSGA-II					
NSGA-II	*HV*	0.55	0.63	0.58	0.01
Random GA		0.34	0.45	0.39	0.01
NSGA-II	*Runtime* (*x*$10^{3}$)	56	70	65	0.5
Random GA		60	77	70	0.5
